# Dual use of e-cigarettes and combustible cigarettes elevates salivary DKK1 levels similar to exclusive smoking

**DOI:** 10.3389/fpubh.2026.1951458

**Published:** 2026-10-06

**Authors:** Nayeli Itzel Vázquez-López, Ramcés Falfán-Valencia, Leslie Chavez-Galan, Ingrid Fricke-Galindo, Espiridión Ramos-Martínez, Raúl Sansores, Alejandra Ramirez Venegas, Cristina Rivera-González, Gloria Pérez-Rubio

**Affiliations:** 1 Pneumogenomics Laboratory, Instituto Nacional de Enfermedades Respiratorias Ismael Cosío Villegas, Mexico City, Mexico; 2 Laboratorio de Inmunología Integrativa, Instituto Nacional de Enfermedades Respiratorias Ismael Cosío Villegas, Mexico City, Mexico; 3 Facultad de Medicina, Unidad de Medicina Experimental, Universidad Nacional Autónoma de México, Mexico City, Mexico; 4 Médica Sur Clinic & Foundation, Mexico City, Mexico; 5 Escuela Militar de Medicina, Universidad del Ejército y Fuerza Aérea, Mexico City, Mexico

**Keywords:** cell death, DKK1, dual electronic-cigarette, Hsp70, inflammation

## Abstract

**Background:**

Animal models and *in vitro* studies have demonstrated that the use of electronic cigarettes causes inflammation, oxidative stress, and cell death. This study aimed to quantify molecules associated with cell death in the saliva of dual electronic cigarette users and to compare these levels with those observed in users of combustible cigarettes.

**Methodology:**

We included three groups: (1) e-cigarette users who additionally smoke combustible cigarettes, “dual users” (DU users, *n* = 56), combustible cigarette smokers (CCS, *n* = 23), and non-smokers (NS, *n* = 40). We evaluated 10 molecules using a multiplex assay.

**Results:**

Dual users of e-cigarettes showed elevated cotinine levels and higher scores on the Fagerström Test for Nicotine Dependence (FTND), indicating greater nicotine dependence compared to combustible cigarette smokers. The dual-user group had significantly higher levels of Dickkopf-1 (DKK1) than the non-smoker group (220.6 pg./mL vs. 0, *p* = 0.030). Positive correlations were identified between DKK1 and 70-kilodalton heat shock protein (HSP70) (rho = 0.298, *p* = 0.042). A negative correlation was also found between HSP70 and the age of vaping initiation (rho = −0.289, *p* = 0.043), and a positive correlation with the duration of vaping sessions (rho = 0.292, *p* = 0.042), indicating that starting vaping at a young age and prolonging sessions may increase the expression of this protein.

**Conclusion:**

Dual users of electronic cigarettes exhibit elevated salivary DKK1 levels and significant correlations with HSP70; these results suggest that chronic use may alter the inflammatory process. The pattern of e-cigarette consumption and the age of initiation modify salivary HSP70 levels. These proteins are involved in cellular stress and inflammatory mechanisms.

## 1 Introduction

Although the use of electronic cigarettes is prohibited in Mexico, consumption is on the rise, especially among teenagers and young adults (1). The health consequences of chronic use of these electronic nicotine delivery systems (ENDS) are still unknown. However, nicotine addiction is a complex and multifactorial disease in which genetic factors, along with the social environment, contribute significantly to the continued use of e-cigarettes (2). Unlike combustible cigarettes, e-cigarettes vaporize a liquid solution composed of nicotine, propylene glycol (PG), vegetable glycerin (VG), and flavorings. In young e-cigarette users from the BAMSE cohort (a prospective birth study based on the Swedish population), no impairment of lung function has been observed; however, there is a higher prevalence of symptoms such as cough and mucus production and greater obesity among e-cigarette users (3). The use of electronic cigarettes has been associated with significant reductions in lung function and respiratory muscle strength (4). Data on the underlying mechanisms show that nicotine-induced sympathetic activation, oxidative stress, and vascular damage are key biological pathways explaining these effects (5).

E-cigarette aerosols generate reactive oxygen species (ROS). These increase oxidative stress, cause DNA damage, mitochondrial dysfunction, and epigenetic alterations (6). Consequently, cell signaling pathways associated with activation of cell death and chronic inflammation are activated (7, 8). In human and murine epithelial cells, ENDS aerosol has been shown to induce programmed cell death mediated by caspase-3/7 (9). *In vitro* assays with gingival and endothelial cells exposed to e-cigarettes showed increased apoptosis, induced via caspase-3 (10). These cell death processes have been explored in greater depth using combustible cigarette smoke. An *in vitro* study of combustible cigarette smoke condensate on human arterial and venous endothelial cells showed that at concentrations below 50 μg/mL, cells exhibit apoptotic-like signaling; however, at higher concentrations (100 μg/mL), it leads to programmed necrosis (11). Another study showed that in A549 cells, 4 h of cigarette smoke exposure induced early apoptotic markers (Bax-α and cytochrome c); however, 48 h after exposure, apoptosis decreased, suggesting greater tissue damage due to downregulation of apoptosis and increased necrosis (12).

The oral cavity is the first point of contact with cigarette smoke in the human body. Evidence shows that smoking is a risk factor for the development and progression of periodontal diseases and precancerous conditions in the oral cavity (13, 14). E-cigarettes can induce oxidative stress in gingival and periodontal tissues (15). In e-cigarette users, lipid peroxidation products increase with oxidative stress, thereby disrupting lipid balance in the oral cavity (16).

In 2023, e-cigarette use was common among adolescents and young adults in the United States (17); however, by 2024, the combined use of e-cigarettes and conventional cigarettes had increased (18). This consumption pattern, known as dual use, is highly prevalent among Caucasian, Asian, and Latin American populations (19, 20). Significantly higher concentrations of biomarkers of exposure to nicotine and toxic substances have been detected in dual users compared with those who use only one product (21). Although the health effects of combustible cigarettes and e-cigarettes have been studied separately, it is important to understand the effects of dual use. This study aimed to quantify cell death molecules in the saliva of dual users of electronic cigarettes who also smoke combustible cigarettes (dual users) and to compare these findings with those of combustible cigarette users.

## 2 Materials and methods

### 2.1 Study population

This comparative observational study included 3 groups: (1) dual users (DU, *n* = 56), combustible cigarette smokers (CCS, *n* = 23), and non-smokers (NS, *n* = 40). We described the study population in detail previously (22). This research was conducted in strict compliance with current ethical regulations for research involving human subjects. The study followed the principles of the Declaration of Helsinki, and the Research Ethics Committee of the Instituto Nacional de Enfermedades Respiratorias Ismael Cosío Villegas (INER) in Mexico City approved all procedures (Protocol B26-20).

Participants were recruited from universities in Mexico between 2022 and 2025. Participation was voluntary, and all individuals were over 18 years of age. Participants’ spirometry results fell within expected parameters for their age, sex, and population. The DU group comprised users who had consumed nicotine-containing electronic cigarettes for at least the preceding 6 months, whereas the CCS group included individuals who had smoked at least one combustible cigarette for 6 months or longer. Exclusion criteria included a history of cardiovascular or autoimmune diseases, asthma, allergies, liver disorders, diabetes, oral diseases or infections, hypertension, pregnancy, bronchial hyperreactivity, cancer, metabolic disorders, chemotherapy, and the use of other substances such as marijuana, cocaine, or alcohol. Nicotine dependence was assessed using a modified version of the Fagerström Test for Nicotine Dependence (FTND). The same questionnaire was administered to both DU and CCS groups, with the term “tobacco” replaced by “electronic cigarette” as appropriate. We used total scores to classify dependence levels: 0–3 points indicated no dependence, 4–8 low dependence, 9–12 moderate dependence, and ≥13 high dependence (23).

### 2.2 Saliva sample collection and cotinine quantification

The participants were instructed to refrain from eating, drinking, vaping, and smoking for at least 2 hours before sample collection. We collected saliva samples by stimulating salivary flow to evaluate cotinine and cell-death molecules in saliva. The inclusion and exclusion criteria were described previously (22). Saliva was collected in a 50 mL polypropylene sterile centrifuge tube. The samples were centrifuged at 4500 rpm for 5 min, separated using micropipettes, and stored at −80 °C until analysis.

Human cotinine in 100 μL samples was quantified by competitive enzyme-linked immunosorbent assay (ELISA) following the manufacturer’s protocol (catalog no. MBS720957; MyBioSource, San Diego, United States). The assay sensitivity is 1.0 ng/mL. Absorbance was measured at 450 nm. All standards and samples were analyzed in duplicate. The mean well value was recorded in ng/mL.

### 2.3 Cell death assessment in saliva samples

To assess cell death in saliva samples, we selected two panels: the ProcartaPlex 4-plex human cell death panel (Catalog Number EPX040-15843-901, Invitrogen, Thermo Fisher Scientific, Waltham, Massachusetts, United States). This panel includes prominent marker proteins involved in cell death. Damage-associated molecular patterns (DAMPs) are released in response to cellular stress [e.g., heat shock 70 kDa protein (HSP70), heat shock 90 kDa protein (HSP90)] and play important roles in disease development and immunogenic cell death (e.g., high mobility group protein B1, HMGB1). The panel also includes Dickkopf-related protein 1 precursor (DKK1), a secreted protein that regulates cell survival through the WNT signaling pathway. To assess cell death via apoptosis, the ProcartaPlex 6-plex human apoptosis panel (Catalog Number EPX06A-15823-901, Invitrogen, Thermo Fisher Scientific, Waltham, Massachusetts, United States) was employed. This panel includes the apoptosis regulator Bcl-2, caspase-3 (active), cleaved poly(ADP-ribose) polymerase (PARP), cytochrome C, glyceraldehyde-3-phosphate dehydrogenase (GAPDH), and cellular tumor antigen p53. In both cases, we used the panels as recommended by the manufacturer. We recorded each molecule’s concentration in pg./mL.

### 2.4 Statistical analysis

Categorical variables were described with frequencies and percentages; numerical variables were described with means (SD) or medians and first and third quartiles as appropriate. The data were recorded in a spreadsheet for subsequent analysis using JASP v0.95.3 (24). Nonparametric statistics were used to compare variables between study groups. The Mann–Whitney *U* test or the Kruskal-Wallis test was employed. Spearman correlation analysis was performed for the continuous numerical variables of molecules of necrosis or apoptosis and the pattern of electronic cigarette consumption. The *p*-value was obtained using a multiple-testing correction based on the false discovery rate (FDR) estimate.

## 3 Results

One hundred nineteen participants were included, divided into three groups: DU (*n* = 56), CCS (*n* = 23), and NS (*n* = 40). The NS group was the oldest, with a median age of 24 years, compared with 21 years for the CCS group and 20 years for the DU group. The group of dual users and exclusive combustible cigarette users had a higher proportion of men compared to the group of non-users, which had a higher proportion of women. By body mass index (BMI), all groups were within the normal weight range, with the NS group the most fit (median 22.0, IQR 20.5–24.0), while subjects in the DU and CCS groups were at the upper end of normal weight (25.4 and 25.8, respectively). However, we found no statistically significant differences. Age at onset was similar between tobacco smokers and dual users (18 years), as were years of consumption (2 years), with no statistically significant differences (*p* > 0.05) between the DU and CCS groups.

Determination of variables related to nicotine addiction by FTND score was 5-fold higher in dual users than in the conventional combustible cigarette group (5.5 vs. 1.0, respectively; *p* = 0.014). Salivary cotinine levels were higher in the DU group compared with the CCS group (39.0 ng/mL vs. 12.0 ng/mL, respectively; *p* = 0.010) (Table 1).

**Table 1:** Characteristics of the study population.

Variable	DU (*n* = 56)	CCS (*n* = 23)	p*	NS (*n* = 40)	p**
Age (years)	20 (19–21)	21 (19–22)	0.092	24 (23-24)	**<0.001**
Sex, men, *n* (%)	25 (44.6)	12 (52.1)	0.423	3 (7.5)	**0.002**
BMI (kg/m^2^)	23.4 (21.8–25.4)	24.5 (21.7–25.8)	0.965	22.0 (20.5–24.0)	0.258
Age at onset (years)	18 (17–20)	18 (16–18)	0.289	—	—
Consumption (years)	2 (1–3)	2 (1–3)	0.280	—	—
Fagerström test for nicotine dependence	5.5 (1.7–9.0)	1.0 (1.0–2.0)	**0.014**	—	—
Cotinine (ng/mL)	39 (11–62)	12 (0–32)	**0.010**	0 (0–11)	**0.010**

DU, dual e-cigarette users; CCS, combustible cigarette smokers; NS, non-users and non-smokers of electronic cigarettes; BMI, body mass index. *p-value by Mann–Whitney *U* test between DU and CCS. **p-value by Mann–Whitney *U* test between DU and NS. Text in bold indicates significant statistical *p*-values.

### 3.1 Levels of molecules of cell death in saliva

We assessed 10 molecules covering the two primary pathways involved in cell death. Among the four molecules evaluated for necrosis (DKK1, HMGB1, HSP70, and HSP90), only HMGB1 was undetectable across the three groups. Among the six-plex panel evaluating apoptosis (BCL2, Caspase-3, Cleaved-PARP, Cytochrome-C, GAPDH, and p53), Caspase-3 and Cleaved-PARP had the lowest values across the three groups.

Overall, molecular levels of cell death in the DU group were higher than in the CCS group for HSP90, Bcl2, cytochrome C, and GAPDH; however, no statistically significant differences were observed (Table 2). When comparing the DU and NS groups, a significant difference in DKK1 levels was observed, with higher levels in the DU group (220.6 pg./mL vs. 0 in the NS group; *p* = 0.003; *p*-value by FDR = 0.030) (Figure 1).

**Table 2:** Molecules of cell death in the saliva of participants included in the study.

Protein (pg/mL)	DU (*n* = 56)	CCS (*n* = 23)	p*	NS (*n* = 40)	p**
DKK1	220.6 (50.1–380.2)	220.6 (0–317.1)	0.674	0 (0–37.5)	**0.030**
HMGB1	0	0	0.674	0 (0-0)	0.683
HSP70	0 (0–131.8)	90.5 (0–131.8)	0.642	0 (0-0)	0.400
HSP90	59.5 (51.8–59.5)	51.8 (51.8–59.5)	0.674	51.8 (43.9–257.9)	0.768
BCL2	14.2 (8.2–21.4)	12.1 (12.1–26.8)	0.674	12.1 (12.1–12.1)	0.518
Caspase-3	0.2 (0.1–0.3)	0.1 (0.1-0.2)	0.500	0.4 (0.4-0.4)	0.400
Cleaved-PARP	0.1 (0.1-0.2)	0.4 (0.4-0.4)	0.500	0 (0-0.1)	0.400
Cytochrome-C	398.4 (181.3–888.7)	134.6 (130.6–138.7)	0.671	935.7 (935.7–1138.7)	0.400
GAPDH	134.6 (130.6–138.7)	124.4 (105.3–143.4)	0.642	117.0 (117.0–117.0)	0.542
p53	5.5 (5.5-5.5)	9.6 (9.6-9.6)	0.642	5.5 (5.5-5.5)	1.000

DU, dual e-cigarette users; CCS, combustible cigarette smokers; NS, non-users and non-smokers of electronic cigarettes. *p-value by multiple testing correction via false discovery rate (FDR) estimation between DU and CCS. **p-value by multiple testing correction via false discovery rate (FDR) estimation between DU and NS. Text in bold indicates significant statistical *p*-values.

**Figure 1:**
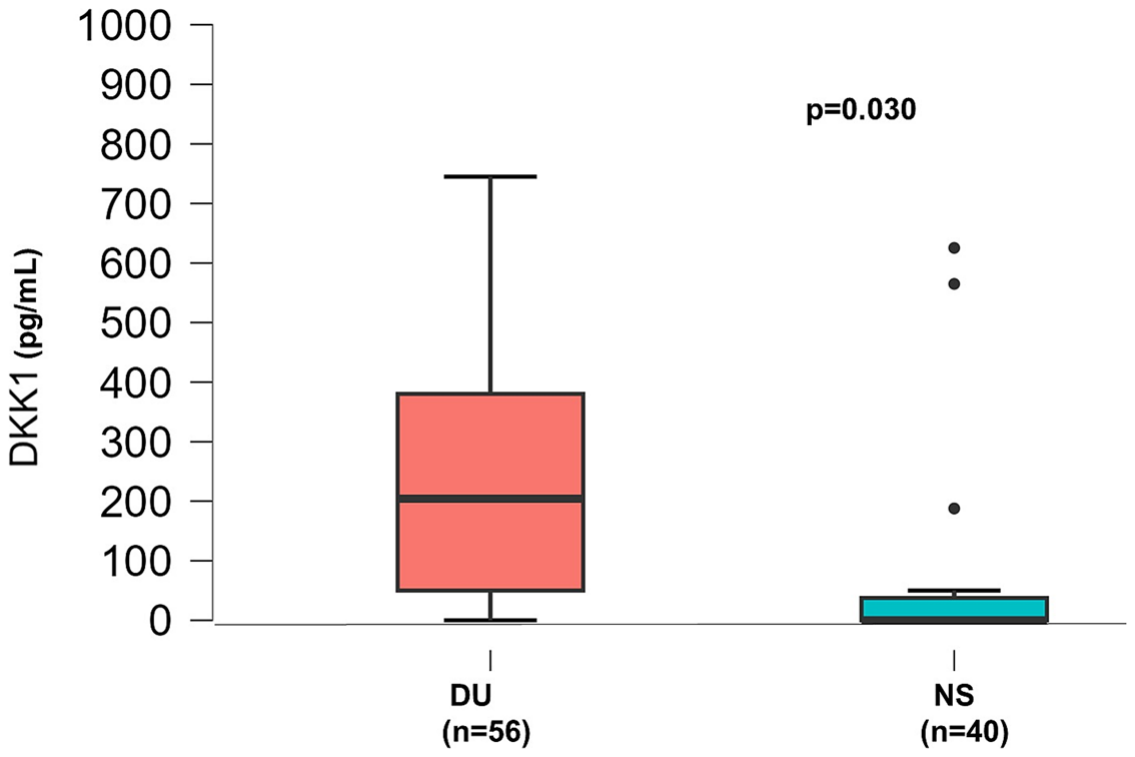
Levels of DKK1 in the saliva of DU and NS groups. DU, dual e-cigarette users; NS, non-users and non-smokers of electronic cigarettes; DKK1, Dickkopf-related protein 1 precursor. *p-value by FDR.

We performed a correlation analysis between the DU group and the analyzed proteins. We found a weak but significant correlation between DKK1 and HSP70 (rho = 0.298, FDR-adjusted *p*-value = 0.042). On the other hand, we performed a correlation analysis between cell death markers and e-cigarette use patterns. We found a weak negative correlation between HSP70 and age at e-cigarette use onset (rho = −0.289, FDR-adjusted *p*-value = 0.043). Finally, HSP70 levels were positively correlated with vaping session duration in minutes (rho = 0.292, FDR-adjusted *p*-value = 0.042) (Figure 2).

**Figure 2:**
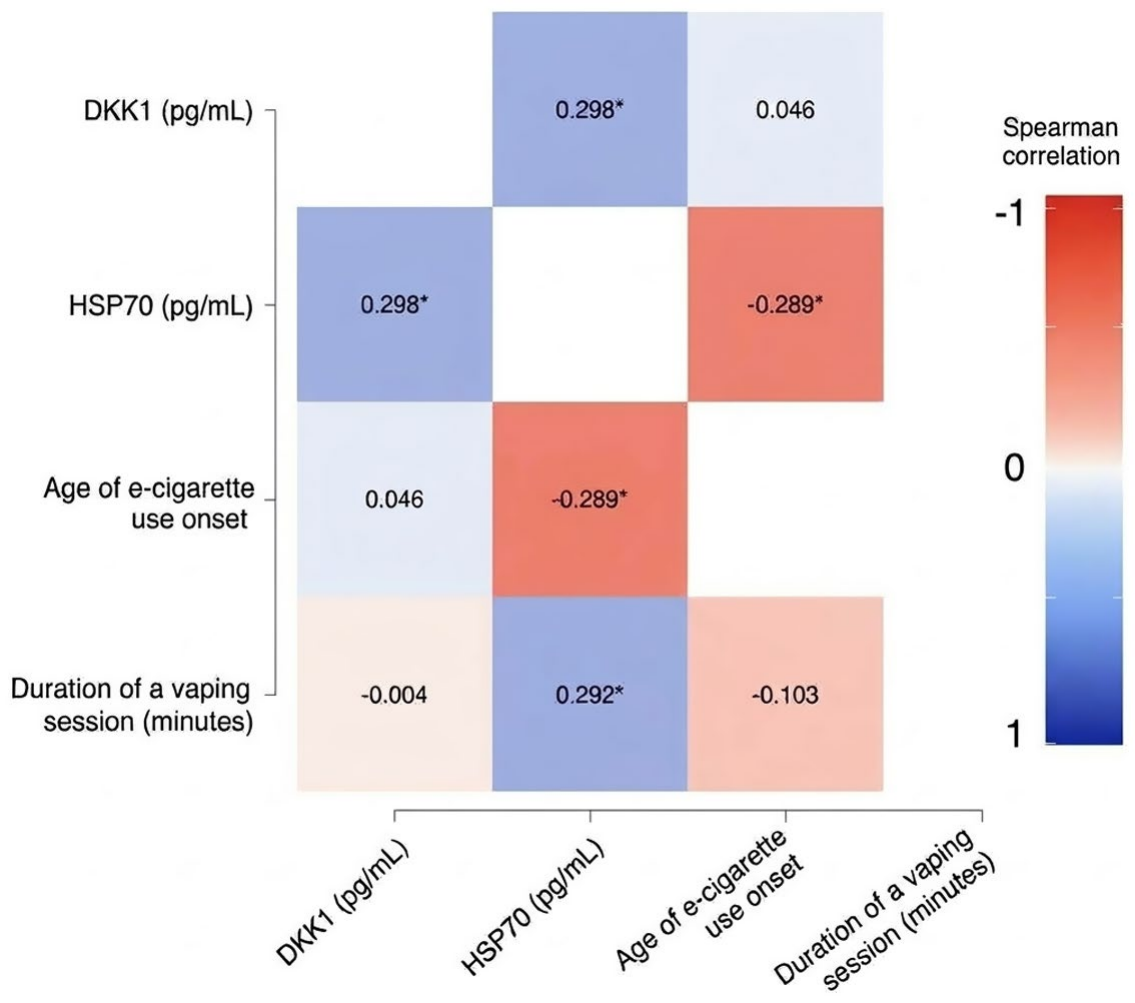
Spearman correlation in dual e-cigarette users (DU group). Statistically significant correlations by FDR (*p* < 0.05) are shown with an asterisk.

## 4 Discussion

Smoking is a risk factor for developing lung diseases such as cancer and chronic obstructive pulmonary disease (COPD). Currently, the use of e-cigarettes in combination with combustible cigarettes complicates the research landscape. In this study, we included dual e-cigarette users to evaluate levels of markers of damage in the oral cavity. Among the markers evaluated, DKK1 levels were higher in the DU group than in the NS group. DKK1 levels were the same between the DU and CCS groups.

DKK1 inhibits the Wnt/β-catenin signaling pathway, which is necessary for osteoblast differentiation and bone formation (25). DKK1 in the lung regulates WNT-induced epithelial cell proliferation (26). In this regard, it was recently reported that pulmonary artery endothelial cells under hypoxic conditions secrete DKK1, which initiates a feedback loop in which DKK1 upregulates cytoskeleton-associated protein 4 (CKAP4) expression and activates the NF-κB pathway, promoting cell proliferation and oxidative stress (27). Thus, the DKK1/CKAP4 axis is recognized as a cell proliferation signal; reports indicate that in patients with chronic periodontitis who smoke, DKK1 expression is higher than in non-smoking subjects (28). DKK1 overexpression has been reported in multiple myeloma, hepatoblastomas, Wilms’ tumors, breast cancer, lung cancer, and esophageal cancer (29, 30). DKK1 has been proposed as an indicator of microenvironmental changes that favor neoplastic cell development (31). However, its role in mediating cell death is less clear, though some reports suggest that DKK1 may have tumor-suppressive and apoptosis-inducing effects independent of canonical Wnt signaling (32).

Previous studies have shown that DKK1 contributes to airway inflammation. In the lungs, DKK1 promotes macrophage and neutrophil infiltration by inhibiting the WNT pathway (33) and induces the production of IL-4, IL-10, and IL-13, as well as Th2 cytokines (34). However, in subjects with COPD, DKK1 levels are lower than in the control group. While COPD is an inflammatory disease, in which elevated DKK1 levels would be expected, the contradictory results reported in COPD are not surprising, as DKK1 function has been reported to depend on the microenvironment in which it is found, as well as on the type of cells and pathological processes being examined (26). Cigarette smoke extract and electronic cigarette vapor contain high levels of ROS, aldehydes, and acrolein. Exposure to these toxins induces inflammation in the oral microenvironment. Inflammatory cytokines such as TNF-alpha, IL-6, and IL-8 promote DKK1 secretion by human gingival fibroblasts and oral epithelial cells as part of the tissue stress response (27).

Importantly, we observed no statistically significant differences in established apoptosis-related markers, including active caspase-3, cleaved PARP, cytochrome C, BCL2, and p53. These findings suggest that the observed biomarker pattern does not provide direct evidence of activation of canonical apoptotic pathways in dual users. However, elevated DKK1 levels suggest an inflammatory process in the oral cavity similar to that observed in combustible cigarette users.

We found that the group of dual e-cigarette users showed a positive correlation between DKK1 and HSP70 levels. HSP70, when released by apoptotic and necrotic cells, is considered a DAMP (35). In the present study, the correlation between DKK1 and HSP70 could indicate an oral microenvironment characterized by increased oxidative stress and the onset of inflammatory processes. However, although HSP70 levels did not differ statistically between the study groups, follow-up is warranted in the dual e-cigarette user group. In addition, high salivary levels of HSP70 were observed when vaping began at a young age (negative correlation), and there is a positive correlation between this protein and the duration of the vaping session (minutes). Although we detected correlations between HSP70 and dual use, HSP70 levels did not differ significantly among the study groups; therefore, they should be interpreted with caution as potential indicators of cellular stress.

Tobacco smoking induces HSP70 expression. *In vitro* assays demonstrate that cigarette smoke extract and extracellular HSP70 induce ATP release and NLRP3 inflammasome activation (36). Notably, DAMPs contribute to inflammation by activating and recruiting various inflammatory cells. It has been reported that in patients with asthma, sputum HSP70 levels are elevated and negatively correlated with forced expiratory volume in 1 second (FEV1) (37). *In vitro* models show that HSP70, in combination with cigarette smoke extract, induces IL-6 and IL-8 production and activates immune cells through toll-like receptors (TLR) 2 and TLR-4 (38).

Our study has limitations, including the sample size and the absence of a group of users who exclusively consume electronic cigarettes. The lack of regulations regarding the manufacture and distribution of these products prevents the characterization and quantification of toxic substances present in electronic cigarettes during use. Based on existing literature, we present a hypothetical model describing the potential biological pathways associated with dual exposure to e-cigarettes and combustible cigarettes and the release of DKK1 (Figure 3). However, we propose monitoring dual users to determine whether chronic use increases extracellular DKK1 and whether this, in turn, may foster an inflammatory environment that activates the inflammasome pathway in this population, given that *in vitro* models have demonstrated this activation mechanism (39). Our results indicate that electronic cigarettes, when used in combination with combustible cigarettes, alter levels of DKK1—a protein involved in critical molecular pathways linked to inflammation.

**Figure 3:**
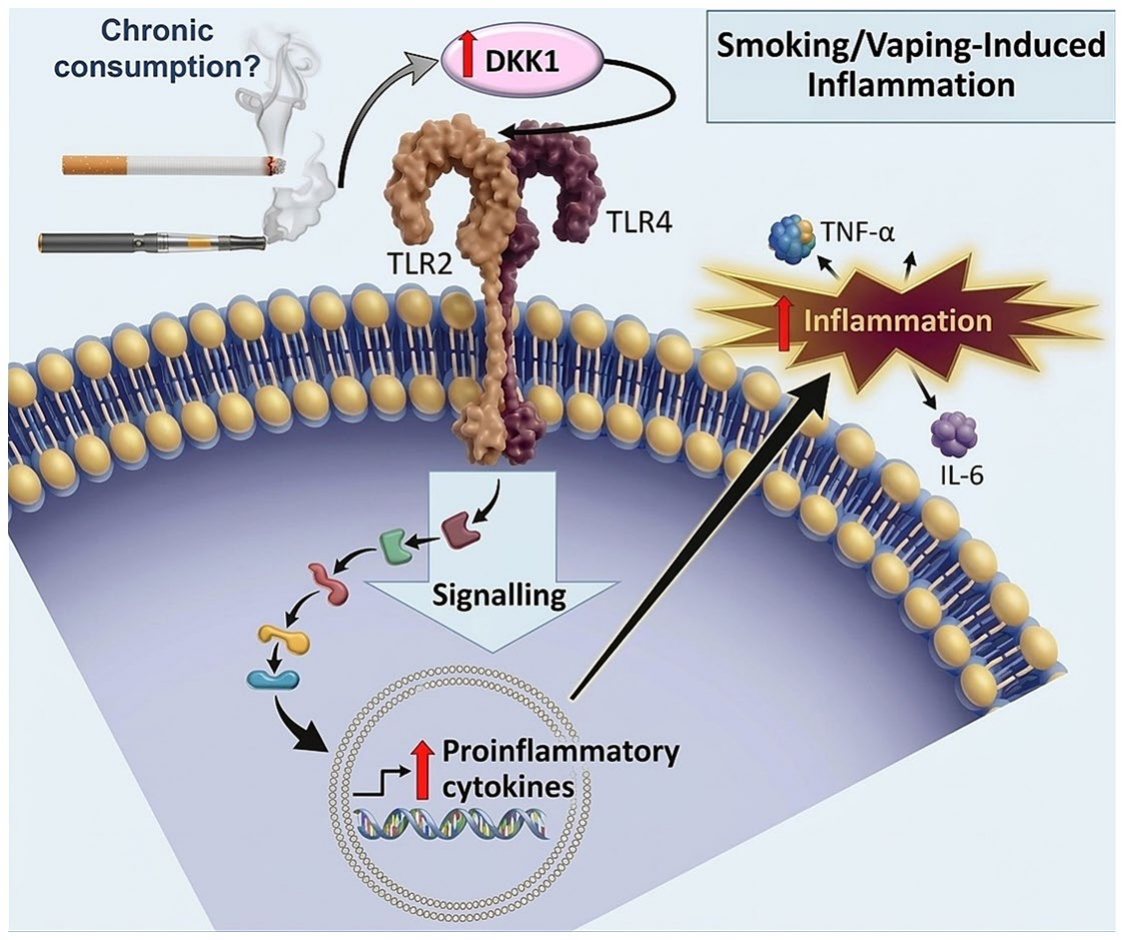
Hypothetical model derived from previous literature describing potential biological pathways associated with dual exposure to electronic and combustible cigarettes. Images provided by Servier Medical Art (https://smart.servier.com) and NIAID NIH BioArt (https://bioart.niaid.nih.gov/).

## Data Availability

The raw data supporting the conclusions of this article will be made available by the authors, without undue reservation.
